# Characterization of Ovarian Steroid Patterns in Female African Lions (*Panthera leo*), and the Effects of Contraception on Reproductive Function

**DOI:** 10.1371/journal.pone.0140373

**Published:** 2015-10-13

**Authors:** Sarah B. Putman, Janine L. Brown, Ashley D. Franklin, Emily C. Schneider, Nicole P. Boisseau, Cheryl S. Asa, Budhan S. Pukazhenthi

**Affiliations:** 1 Smithsonian Conservation Biology Institute, Front Royal, Virginia, United States of America; 2 George Mason University, Fairfax, Virginia, United States of America; 3 Point Defiance Zoo and Aquarium, Tacoma, Washington, United States of America; 4 Shady Grove Fertility, Rockville, Maryland, United States of America; 5 AZA Wildlife Contraception Center, St. Louis, Missouri, United States of America; Federal University of Parana (UFPR)—Campus Palotina, BRAZIL

## Abstract

Because of poor reproduction after the lifting of an 8-year breeding moratorium, a biomedical survey of female lions in U.S. zoos was initiated in 2007. Fecal estrogen (FEM), progestagen (FPM) and glucocorticoid (FGM) metabolites were analyzed in samples collected 3–4 times per wk from 28 lions at 17 facilities (0.9–13.8 yr of age) for 4 mo—3.5 yr and body weights were obtained ~monthly from 17 animals at eight facilities (0.0–3.0 yr of age). Based on FEM, estrous cycle length averaged 17.5 ± 0.4 d in duration, with estrus lasting 4.4 ± 0.2 d. All but one female exhibited waves of estrogenic activity indicative of follicular activity; however, not all females expressed estrous behaviors (73%), suggesting silent estrus was common. Female lions experienced puberty earlier than expected; waves of estrogenic activity were observed as young as 1.1 yr of age, which may be related to a faster growth rate of captive vs. wild lions. Mean gestation length was 109.5 ± 1.0 d, whereas the non-pregnant luteal phase was less than half (46.0 ± 1.2 d). Non-mating induced increases in FPM were observed in 33% of females housed without a male, consistent with spontaneous ovulation. A number of study animals had been contracepted, and the return to cyclicity after treatment withdrawal, while variable, was ~4.0 yr and longer than the 1-yr expected efficacy, especially for those implanted with Suprelorin. For FGM, there were no differences in overall, baseline or peak mean concentrations among the age groups or across seasons, nor were there any relationships between reproductive parameters and FGM concentrations. Overall, results suggest that poor reproduction in lions after the breeding moratorium was not related to altered adrenal or ovarian steroid activity, but for some females may have been a consequence of individual institutions’ management decisions.

## Introduction

Globally, African lions (*Panthera leo*) are listed as vulnerable with a decreasing population trend [1], although in western and central Africa, lions are considered endangered [2]. In 2004, the total number of lions in Africa was estimated to be 16,000–30,000 individuals, a reduction of >97% over a 200-year period [3]. Today, only seven countries in Africa have more than 1,000 lions [4] and most populations exist only in small areas of dry forests, grasslands and protected reserves [5,6]. Pressures from persecution [7,8], disease [9,10] and habitat loss [11] are main causes of wild lion population declines. Conserving this species long-term likely will require improved *in situ* management in combination with establishing e*x situ* ‘insurance’ populations as part of overall conservation efforts [12,13]. Natural mating is an important aspect of captive breeding, but when that fails or more intensive genetic management is needed, zoos often rely on assisted reproductive technologies (ART), like artificial insemination (AI) and *in vitro* fertilization (IVF). To do so, a thorough knowledge of species reproductive mechanisms is required so that species-specific protocols can be developed [14–17].

Female lion reproductive biology appears to be similar to other *Felidae* in that females exhibit variable estrous cycle lengths, non-pregnant luteal phases (NPLP) that are shorter than the length of a pregnancy, and they are considered induced ovulators but can ovulate spontaneously [18]. Historically, African lions have bred relatively well in captivity [19–21], especially when compared to other felids [22–26]. As a result, reproduction has often exceeded available space. In one instance, between ~1990 and 1998, the North American African Lion Species Survival Plan (SSP) program instituted a breeding moratorium to better manage animal spaces, as well as to remove African/Asiatic hybrids and individuals of unknown pedigree from the breeding population. New founders were imported from Africa to bolster the genetics of the population. However, after the moratorium was lifted in 1999, the captive population experienced a 6-year period of low fecundity. Only 35.4% of reproductive-age females produced offspring between 1999 and 2005, compared to 68% between 1990 and 1998 [27] when breeding was limited to new founders. Thus, in 2007, the SSP requested a reproductive assessment of both male and female lions, including gonadal hormone activity, to determine underlying causes of poor reproduction.

At that time, surprisingly little was known about lion endocrine function. The majority of extant studies were based on behavioral observations only [28,29]. Few had collected biological data and these involved only a small number of captive animals [30,31], post-mortem examinations of culled individuals in the wild [32] or short-term (< 3 hr) blood sampling [33]. With the advent of non-invasive hormone monitoring to study wildlife endocrinology, including identifying puberty onset, assessing reproductive seasonality, diagnosing pregnancy, and determining the effect of management on reproductive success [34–36], our laboratory initiated a study to use longitudinal fecal hormone analyses to understand possible causes of poor reproduction in female lions after the breeding moratorium.

We also assessed the impact of contraception, an approach used during the moratorium, on ovarian function and subsequent re-establishment of cyclicity post-contraception. For more than 25 years, silastic implants containing the synthetic progestin melengestrol acetate (MGA) were the most popular contraception method [37]. Progestins act by thickening cervical mucus to hinder sperm transport, interfering with implantation and preventing the LH surge required for ovulation [38]. However, an increased incidence of endometrial hyperplasia in felids was linked to MGA usage, and long-term treatment (>4 yr) was associated with advanced-grade lesions [39]. Some lions were subsequently treated with another progestin, medroxyprogesterone acetate (Depo-Provera®), although caution was advised with regards to its use following the Munson et al. studies. Two gonadotropin-releasing hormone (GnRH) agonists were then considered as safer alternatives: Depot Lupron® (leuprolide acetate injection, 1-, 3- or 4-mo formulations) and Suprelorin® (lipid-matrix implants containing deslorelin acetate, with minimum duration of efficacy 6 or 12 months) [37]. GnRH agonists bind to receptors with high affinity and cause a prolonged down-regulation of pituitary GnRH receptors, thereby inhibiting gonadotropin secretion [37]. Depot Lupron, MPA, MGA and Suprelorin were all used during the moratorium; however, no studies had evaluated the effect of these contraceptives on subsequent reproductive fitness. Last, because stress is known to negatively impact reproduction via elevated glucocorticoid production [24,40], adrenal activity was also evaluated in these females.

The objectives of this study were to: 1) characterize the reproductive cycle patterns of pre- and post-pubertal female lions through analyses of fecal estrogen (FEM) and progestagen (FPM) metabolites; 2) compare ovarian steroid profiles between pregnant and nonpregnant females; 3) evaluate the influence of contraceptive treatments on steroid hormone production and the resumption of normal ovarian cycles; and 4) determine the relationship between reproductive parameters and fecal glucocorticoid metabolite (FGM) concentrations as an indicator of adrenal activity and stress. These data are essential for determining the reproductive status of individual lions to aid in diagnosing fertility problems, and providing baseline information needed to develop species appropriate management techniques.

## Materials and Methods

### Ethics Statement

The Smithsonian Conservation Biology Institute (SCBI), the Indianapolis Zoo, and the Lincoln Park Zoological Gardens Animal Care and Use Committee approved this project. None of the other participating institutions required additional approval since fecal samples were collected non-invasively as part of routine animal care. Fecal collections were noninvasive and did not affect the animal’s daily routine. The decision to contracept animals was made by each facility in consultation with the Lion SSP and the AZA Wildlife Contraception Center (WCC) at the Saint Louis Zoo. Body weights were obtained from animals as part of a zoo’s routine management protocol.

### Animals and sample collection

A total of 38 female lions at 19 facilities, ranging from <1 mo to 13.8 yr of age, were used in one, two or all three components of this study [S1 Table; reproductive assessment (n = 22), contraception evaluation (n = 11) and body weight measurement (n = 17)]. All animals had indoor and outdoor access daily, provided that outdoor temperatures exceeded -1°C. All animals were visible to the public at least 1 h and up to 24 h every day. The diet composition varied by facility with approximately half of the zoos feeding a combination of horse meat and beef, while others fed strictly horse meat or beef, with Nebraska Brand (Central Nebraska Packing, Inc., North Platte, NE) and Natural Balance (Natural Balance Pet Foods, Burbank, CA) being the manufacturer of 85% of the diets. Water was available ad libitum and on average, individuals were fasted up to 1 d a week and provided bones 2 d a week. As part of animal management, 11 females were contracepted with either Suprelorin (N = 8), MGA (N = 2) or Lupron (N = 1) during all or part of the study period.

Fecal samples were collected within 24 h of being voided, 3–4 d/wk for 4 mo to 3.5 yr (S1 Table, N = 28). No special permits were required for sample collection since all animals were managed in captivity within various zoos in North America and the project was approved by the Lion Species Survival Plan. Samples were stored frozen until overnight shipment to the endocrinology laboratory at SCBI for processing and steroid hormone analysis. Behavioral data from keeper records, including observations of estrus behaviors, such as lordosis, rolling and solicitation, male interest and breeding attempts were available for 11 of the study animals. Dates of parturition were verified using the studbook [27].

Body weights were obtained from 17 individuals at least once a month for the first 12 mo of age, at which point the duration of weight measurement collection varied by facility (S1 Table). To weigh young cubs (≤ 4 mo), the weight of a plastic tub was tared from a platform balance, the cub was placed inside the tub and the weight was recorded. Older individuals (> 4 mo) were weighed during training sessions using platform scales placed in the animal’s indoor enclosure or in a squeeze cage.

### Fecal steroid hormone processing and analysis

Fecal samples were processed and hormone metabolites extracted as previously described [34]. Briefly, samples were freeze-dried in a lyophilizer (VirTis Ultra 35XL, SP Scientific, Warminster, PA) for 5 d, crushed to a powder, sifted, and stored in polypropylene tubes at -20°C. Samples were weighed (0.20 ± 0.02 g) into 16x125 mm glass tubes (Fisherbrand, Thermo Fisher, Pittsburgh, PA) to which ~20,000 dpm ^3^H-linked estradiol-17β tracer (NEN Radiochemicals, Perkin Elmer, Boston, MA) was added to determine procedural loss, followed by 5 mL of 90% ethanol:10% de-ionized water. Samples were boiled in a 95°C water bath for 20 min with 100% ethanol added as needed to maintain 5 mL volume, and then centrifuged at 500 x *g* for 20 min. The supernatant was aspirated and 5 mL of 90% ethanol was added to the pellet, and the sample was vortexed (pulse rate 1/s, speed 65; Multi-tube Vortexer; Glas-Col, Terre Haute, IN) for 30 s. Sample extracts were centrifuged for 15 min at 500 x *g*, and the supernatants combined and dried down under forced air. Extracts were reconstituted with 1 mL of 100% methanol (HPLC Grade Methanol, Thermo Fisher, Pittsburgh, PA), dried under forced air and reconstituted with 1 mL of preservative-free phosphate buffer (0.2 M NaH_2_PO_4_, 0.2 M Na_2_HPO_4_, 0.15 M NaCl; pH 7.0). Each sample was vortexed for 15 s and placed in an ultrasonic cleaner water bath (Cole Parmer Instrument Company, Vernon Hills, IL) for 15 min. Sample extracts were further diluted in preservative-free phosphate buffer as needed for each hormone assay: 1:250–1:6,000 for progestagens; 1:20–1:100 for estrogens; and 1:10–1:200 for glucocorticoids. All sample extracts and dilutions were stored in polypropylene tubes at -20°C until analysis. The average fecal extraction efficiency (79.0 ± 0.9%) was based on the recovery of ^3^H-estradiol-17β (~20,000 dpm) added to each sample prior to extraction.

A single-antibody enzymeimmunoassay (EIA) technique was used for hormone analysis as previously described [41]. Assays utilized a monoclonal progesterone antibody (Quidel CL425, C.J. Munro, University of California, Davis, CA) and polyclonal antibodies to estradiol-17β (R4972; C.J. Munro) and cortisol (R4866; C.J. Munro) to quantify FPM, FEM and FGM, respectively. Briefly, antibodies in coating buffer (0.015 M Na_2_CO_3_, #S2127; 0.035 M NaHCO_3_, #S8875, Sigma Aldrich, St. Louis, MO; pH 9.6) were adsorbed to flat-bottomed, high-binding 96-well microtitre plates (Nunc-Immuno, Thermo Fisher) and incubated ≥ 8 h at 4°C. The plates were washed (0.05% Tween 20, #P1379, Sigma Aldrich; in 0.15 M NaCl solution, #S271, Fisherbrand) five times and loaded with 0.05 mL (0.02 mL for estradiol) standards (progesterone: #P0130; β-Estradiol: #E1132; hydrocortisone: #H4001, Sigma-Aldrich) in triplicate, and internal controls and diluted samples in duplicate. Next, 0.05 mL horseradish peroxidase (HRP) solution was added to each well and plates were incubated at room temperature (RT) for 2 h (1 h for cortisol). After incubation, plates were washed five times and 0.1 mL ABTS solution (0.04 M ABTS diammonium salt, #0400, Amresco, Solon, OH; 0.5 M H_2_O_2_, #BP2633, Fisherbrand, in 0.05 M citric acid buffer, #C0759, Sigma Aldrich; pH 4.0) was added to each well. Assays were read on a microplate reader (MRX, Dynex Technologies, Chantilly, VA) at 405 nm (ref. 490 nm) to an optical density (OD) of 1.0 (range 0.9–1.1) for the 0 ng/mL standard.

Estradiol HRP was obtained from UC Davis. Horseradish peroxidase ligands for progesterone and cortisol were prepared at the SCBI. Briefly, Solution A was made by weighing 0.02 mM steroid (progesterone 3-(O-carboxymethyl) oxime, #P3277; or hydrocortisone 3-(O-carboxymethyl) oxime, #H6635, Sigma Aldrich) into a glass 12x75 mm tube (Fisherbrand) with a micro stir bar (8x1.5 mm; Fisherbrand), capped and placed into a beaker of ice on a stir plate. The steroid was dissolved in 0.4 mL N,N-dimethyl formamide (#D4551, Sigma Aldrich) at 0°C, 0.003 mL 4-methylmorpholine (#M56557, Sigma Aldrich) was added while stirring, and the anhydrous reaction was cooled to -15°C, followed by addition of 0.003 mL isobutyl chloroformate (#177989, Sigma Aldrich) and stirring at -15°C for 3 min. Solution B was made by dissolving 40 mg HRP (#P8375, Sigma Aldrich) in 0.4 mL distilled water and 0.3 mL N,N-dimethyl formamide in a 12x75 mm glass tube with a micro stirring bar, which was then placed in a beaker of ice on a stir plate and cooled to 0°C. While stirring Solution B (HRP) at 0°C, Solution A (steroid; kept in a beaker of ice to maintain at 0°C) was slowly pipetted, 0.01 mL at a time, until all of Solution A was combined with Solution B. The reaction mixture was stirred for 1 h at -15°C and then mixed at 0°C for 2 h. Four milligrams NaHCO_3_ (Sigma Aldrich) was fully dissolved in the mixture, which was pipetted into dialysis membrane tubing (Spectrum Spectra/Por 4 RC, 12,000–14,000 Dalton MWCO, 10 mm width; Spectrum Laboratories, Thermo Fisher, Pittsburgh, PA). Double knots were tied at each end and a Spectrum Spectra/Por magnetic, weighted closure (Spectrum Laboratories, Thermo Fisher, Pittsburgh, PA) was attached to the bottom end. The tubing was suspended in a 2 L graduated cylinder (Fisherbrand) containing a stir bar (50x8 mm, Fisherbrand) and filled with distilled water. For 3 d, the graduated cylinder was kept at 4°C on a stir plate and the distilled water was changed twice a day. The dialyzed HRP/steroid mixture was then emptied into a glass 12x75 mm tube.

A Sephadex G-25 (#G25150, medium; Sigma Aldrich, St. Louis, MO) column (Vantage L Lab Column, VL 2.2 x 50 cm, EMD Millipore, Darmstadt, GER) was prepared by equilibration at RT with 500 mL of preservative-free phosphate buffer (0.05 M Na_2_HPO_4_, #S393; NaH_2_PO_4_, #BP329; Fisherbrand; pH 7.5). To remove unconjugated steroid, 10 mL 0.05 M phosphate buffer containing 2% (w/v) BSA (#7500802, Proliant, Boone, IA) was layered onto the column and immediately followed by the HRP/steroid mixture. When the conjugated steroid (indicated as a brown band) neared the bottom, 1 ml aliquots (~25 drops each) were collected into 12x75 mm glass tubes, including at least 5 tubes after the brown band eluted from the column. The tubes containing the HRP-conjugated steroid (aliquots brown in color) were pooled, and 0.25 mL aliquots pipetted into labeled vials, lyophilized to dryness and stored at -80°C. For use, a vial was reconstituted with 0.25 mL distilled water, tapped gently and stored at -20°C.

Assay sensitivities, based on 90% binding were: progesterone, 0.02 ng/mL; estradiol, 0.95 ng/mL; cortisol, 0.08 ng/mL. Intra-assay coefficients of variation (CV) were <10% between sample duplicates and inter-assay CVs for two internal controls analyzed on each assay were: progesterone, 8.1% and 13.7% (N = 255); estradiol, 9.3% and 14.9% (N = 246); cortisol, 6.9% and 9.6% (N = 199), respectively. Hormone data are reported as μg/g feces.

### Hormone assay validations

All assays were validated for lions by demonstrating: 1) parallelism between serial dilutions of fecal extracts and the respective standard curves; 2) significant recovery of steroid standard added to fecal extracts; and 3) biological relevance of hormone data. For parallelism tests, two-fold serial dilutions of samples were analyzed in each EIA (neat to 1:128). Slopes of the standards and sample dilutions, respectively were -12.7 and -14.1 (r = 0.94) for the progesterone, -12.6 and -12.9 (r = 0.99) for the estradiol, and -11.5 and -11.7 (r = 0.99) for the cortisol EIA, respectively. Mass recovery tests were conducted by combining equal volumes of diluted fecal extract and known amounts of exogenous hormone and calculating the difference between the expected and observed concentrations of exogenous hormone (progesterone, y = 0.87x + 2.25, r = 0.99; estradiol, y = 1.11x – 0.08, r = 0.99; cortisol, y = 1.00x + 15.19, r = 0.99) [40,41]. Assays were biologically validated by observations of expected hormonal changes in conjunction with known physiological events: increases in FEM observed in conjunction with behavioral estrus and mating, and FPM concentrations increased during pregnancies (see Results). An immobilization and treatment for an undetermined illness causing a high white blood cell count with symptoms including lethargy and a possible seizure in an individual lioness was utilized to validate the FGM EIA; average FGM in three samples collected over 6 days encompassing the onset of symptoms and treatment (0.57 ± 0.07 μg/g; N = 3) was higher than mean FGM concentrations in samples collected 2 weeks before (0.15 ± 0.02 μg/g; N = 7) and 2 weeks after (0.23 ± 0.03 μg/g; N = 6; *F*_2,14_ = 42.39) treatment (*P* < 0.001).

### High performance liquid chromatography (HPLC)

HPLC analysis (Varian ProStar; Varian Analytical Instruments, Lexington, MA) was performed to determine numbers and proportions of immunoactive steroid hormone metabolites present in lion feces using previously published protocols [42,43], with minor modifications. Three extracted samples for each hormone were combined, filtered (0.2 μm) and evaporated to dryness. Resulting pooled extracts were resuspended in 0.5 mL PBS (0.03 M Na_2_HPO_4_, 0.02 M NaH_2_PO_4_, 0.15 M NaCl, 0.002 M NaN_3_, pH: 5.0), filtered through a C18 Spice cartridge (#01–10, Analtech Inc., Newark, DE), and dried under forced air. Radioactive tracers (~14,000 dpm; ^3^H-estrone sulfate, ^3^H-estradiol 17-β, ^3^H-progesterone, and ^3^H-cortisol) were added to the appropriate pooled sample as chromatographic markers and dried again. The extract was reconstituted in 0.3 mL methanol, sonicated for 5 min, and 0.05 mL was loaded onto a reverse-phase C18 HPLC column (Agilent Technologies, Santa Clara, CA). For progestagens, the sample was separated using a 20–100% linear gradient of acetonitrile:water over 120 min (1 mL/min flow rate; 1 mL fractions). For estrogens and glucocorticoids, the samples were separated using a 20–80% linear gradient of methanol:water over 80 min (1 mL/min flow rate; 1 mL fractions). An aliquot of each fraction was counted on a multi-purpose β-radiation scintillation counter (LS 6500, Beckman Coulter, Brea, CA) and the remainder of each fraction was dried and reconstituted in 0.25 mL preservative-free phosphate buffer for EIA analysis. Each fraction was analyzed in singlet and retention times of the radioactive markers and immunologic activity were compared to identify hormone metabolites.

### Data analyses

Females were separated into three age groups representative of reproductive life stages: subadult, 0.91–2.99 yr (N = 6); adult, 3.00–9.99 yr (N = 11); and aged, 10.00–13.99 yr (N = 5). An iterative process was utilized to calculate baseline concentrations for steroid hormones in each individual animal as described previously [44]. Briefly, all values exceeding the mean plus two times the SD (1.75 times the SD for FPM) were excluded, and the process repeated until no further data points could be removed. The resulting mean was considered the baseline concentration and all values greater than 2 SD (1.75 SD for FPM) above the mean were considered ‘elevated’. Estrous events were classified as a cluster of at least two consecutive samples with elevated FEM concentrations, and the duration of estrus was the number of days FEM concentrations were elevated. Additionally, individual peaks flanked on either side by samples of near threshold FEM concentrations were considered to be estrous events. Estrous cycle lengths were calculated as the number of days between FEM peaks, using the highest concentration in a cluster as the peak. However, intervals of longer than 52 d without an FEM peak were considered anestrous periods [44]. If more than two successive days separated sample collections, the cycle was not included in the analyses. The proportion of cycles in which behavioral estrus was associated with elevated FEM concentrations also was determined. Correlations between peaks in hormone concentrations were determined using Chi-square tests. To compare seasonality, hormone concentrations were first averaged by individual within each season. For each hormone, repeated measures analysis of covariance (ANCOVA) utilizing an AR(1) covariance matrix structure and Tukey multiple mean comparison tests determined the differences in hormone concentration across seasons.

Analysis of covariance with Tukey multiple mean comparison tests were used to compare age group, parity and contraception history differences in estrus duration and estrous cycle length. To calculate the frequency of synchronized estrous cycles, only instances where at least two females at a facility were housed together, both participated in the study and samples were collected within 1 d of each other. By female, the dates of estrous cycles were recorded and then the dates were compared between each female at a facility. When at least 1 d of an estrous cycle overlapped with the other female, then the cycle was considered synchronized.

Luteal phases were defined as > 7 consecutive days of FPM concentrations [44] ≥ 1.75 SD above the mean baseline, and pregnancies were confirmed after 60 d of elevated FPM. To assess differences in FPM concentrations between pregnant luteal phases (PLPs) and non-pregnant luteal phases (NPLPs), weekly averages of FPM were calculated for 3 wk before and 22 wk after ovulation or until the start of another luteal phase. Comparisons in weekly FPM concentration by luteal phase type (PLP, NPLP) were calculated using ANCOVA with post-hoc Bonferroni correction analysis.

One-way analysis of variance (ANOVA) with age group as a fixed factor, and assuming heterogeneous variances with Tukey multiple mean comparison tests, were used to determine differences in average and baseline hormone concentration by age group [45]. All individuals in the aged age group were parous and had been previously contracepted. To insure that this did not impact the results, hormone concentrations and reproductive events were evaluated by parity and contraception history. Differences in hormone concentration between parous and nulliparous individuals and between naïve and previously contracepted females were determined using ANOVA.

For individuals that were contracepted for a portion of the study, only non-contracepted data were used in calculations of individual baseline and mean hormone concentrations. Mean and baseline hormone concentrations during a period of contraception were calculated separately for each individual. Differences in hormone concentrations before and after contraceptive treatment were determined using ANOVA for individuals with only pre- and post-treatment sample collection. Analysis of covariance with Tukey multiple mean comparison tests were used for females with samples collected several months after treatment, as well as pre- and immediate post- treatment sampling. The time from the last contraceptive treatment to when a female gave birth (based on studbook data) was calculated by individual and averaged by treatment group (Suprelorin, MGA, Depot Lupron) for overall efficacy duration.

Body weights of zoo lions were averaged by month of age. Weight data from wild cubs in Kruger National Park were obtained from previously published data [32,46]. Weight differences between wild and captive lions for each month of age were determined using ANCOVA. Unless noted, data are reported as mean ± standard error of the mean (SE) and significances were determined at the *P* < 0.05 α level. To account for repeated sampling within individual animals, any analyses utilizing multiple data points from the same animal (e.g., estrous cycle length) were blocked by individual or calculated with repeated measures and operated with the Kenward-Roger degrees of freedom method. Analyses were conducted using SAS v. 9.3 (SAS Institute Inc., Cary, NC, USA).

## Results

### HPLC analyses

Analysis of HPLC-separated female lion fecal eluates revealed the presence of two estrogen metabolites, the majority of which (89%) co-eluted with the ^3^H-estradiol 17-β tracer, with a more polar peak comprising the remaining 11% of immunoreactivity. By contrast, no progestagen immunoactivity was observed in association with the ^3^H-progesterone tracer. Rather, immunoreactivity was associated with three more polar metabolites representing 41%, 10%, and 35% of the total immunoactivity, and one less polar metabolite with 12% of the immunoactivity. A majority of FGM immunoreactivity (87%) co-eluted with ^3^H-cortisol, with one less polar metabolite accounting for the rest of the immunoreactivity.

### Reproductive and adrenal fecal steroid hormones by reproductive life stage

Mean, baseline and peak FEM, FPM and FGM by reproductive life stage age group are summarized in Table 1. Mean and baseline FEM concentrations across age groups were similar (*F*_2,16_ = 1.38; *P* = 0.28 and *F*_2,16_ = 1.27; *P* = 0.31, respectively), whereas mean peak FEM were lower in the sub-adult group as compared to adults (*F*_2,16_ = 4.21; *P* = 0.03). FPM concentrations across age groups differed for overall mean (*F*_2,16_ = 7.11; *P* = 0.006), baseline mean (*F*_2,16_ = 5.98; *P* = 0.01) and peak mean (*F*_2,16_ = 14.96; *P* < 0.001) (Table 1). The sub-adult age group FPM was consistently lower than the adults (mean, *P* = 0.01; baseline, *P* = 0.03; peak, *P* < 0.001) and differed from the aged animals in overall (*P* = 0.01) and baseline (*P* = 0.02) mean concentrations. No differences in overall mean, baseline or peak mean FGM were observed among the age groups (mean, *F*_2,16_ = 0.25, *P* = 0.78; baseline, *F*_2,16_ = 1.44, *P* = 0.27; peak, *F*_2,16_ = 1.44, *P* = 0.26). Fecal estrogen metabolite concentrations were not correlated with surges in FGM in any of the study animals (X^2^ (1, N = 3689) = 2.89; *P* = 0.09). Furthermore, there were no seasonal changes in reproductive or adrenal hormone patterns (FEM: *F*_3,39.1_ = 1.18, *P* = 0.33; FPM: *F*_3,38.4_ = 0.05, *P* = 0.99; FGM: *F*_3,39.4_ = 0.73, *P* = 0.54).

**Table 1:** Overall, baseline and peak mean (± SE) concentrations of fecal estrogen, progestagen, and glucocorticoid metabolites by age group.

	Fecal Estrogens (μg/g)			Fecal Progestagens (μg/g)			Fecal Glucocorticoids (μg/g)		
Age Group	Overall	Baseline	Peak	Overall	Baseline	Peak	Overall	Baseline	Peak
Subadult	0.23 ± 0.03^a^	0.21 ± 0.02^a^	0.57 ± 0.07^a^	1.37 ± 0.43^a^	1.26 ± 0.42^a^	3.73 ± 0.75^a^	0.14 ± 0.01^a^	0.12 ± 0.01^a^	0.45 ± 0.06^a^
Adult	0.29 ± 0.02^a^	0.24 ± 0.02^a^	0.82 ± 0.08^b^	3.32 ± 0.43^b^	2.75 ± 0.40^b^	11.33 ± 1.25^b^	0.15 ± 0.02^a^	0.13 ± 0.02^a^	0.79 ± 0.16^a^
Aged	0.36 ± 0.07^a^	0.32 ± 0.06^a^	0.77 ± 0.16^a^ ^,^ ^b^	5.36 ± 1.40^b^	4.51 ± 1.12^b^	10.03 ± 2.21^b^	0.17 ± 0.02^a^	0.16 ± 0.02^a^	0.43 ± 0.09^a^

^a,b,c^ Within column, differing letter superscripts indicate a significant difference (*P* < 0.05).

### Estrous cycle patterns

**Fig 1:**
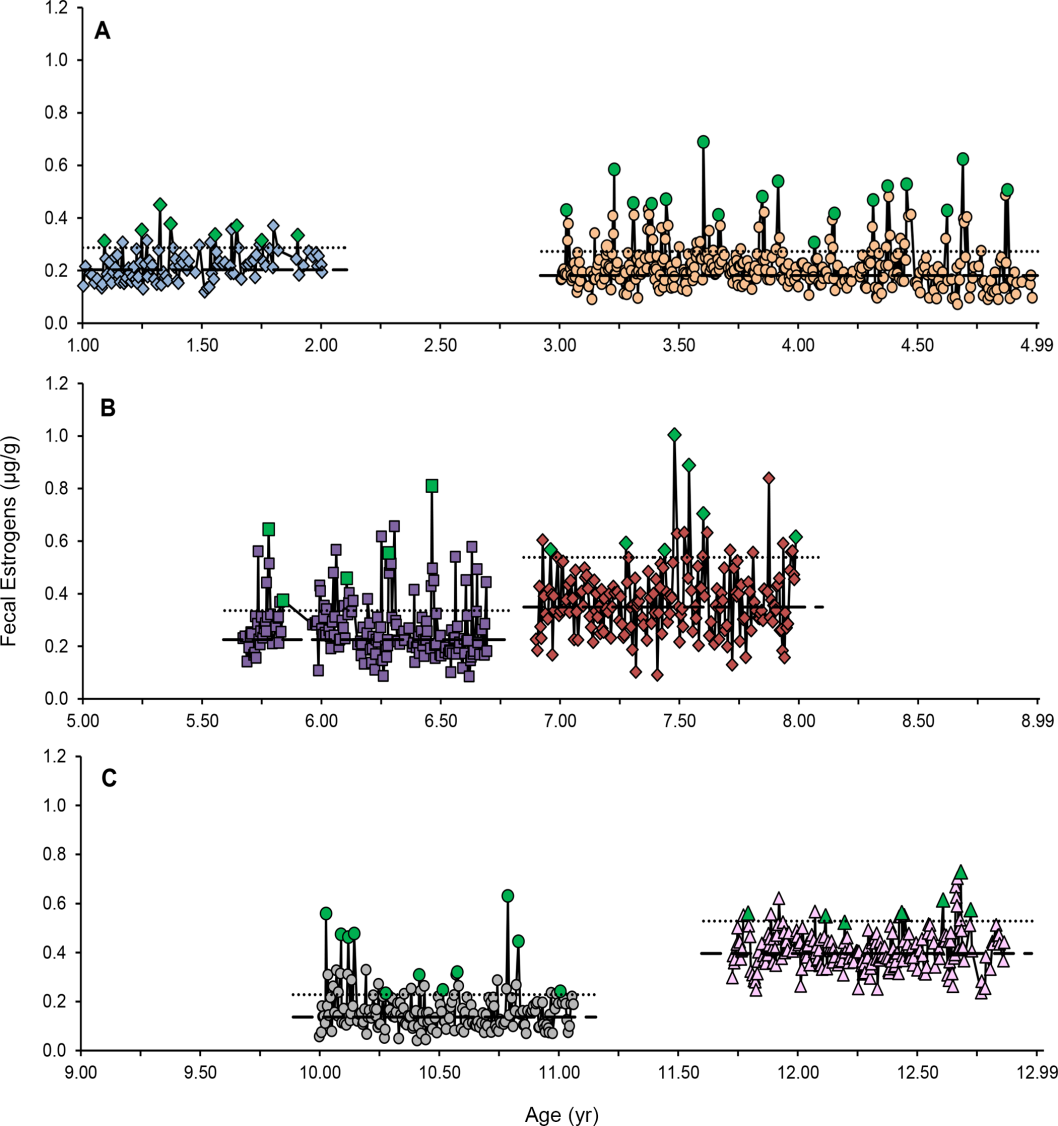
Fecal estrogen metabolite (μg/g) profiles of six females of diverse ages. Panel A: SB408, blue diamonds and SB247, orange circles; Panel B: SB205, purple squares and SB235, red diamonds; Panel C: SB140, grey circles and SB76, pink triangles. Baseline (dashed line) and threshold (dotted line) concentrations were calculated for each female. Estrous peaks are denoted with green data points.

Out of 22 females monitored longitudinally, 21 exhibited estrous cyclicity (i.e., periods of elevated FEM). Cycles were observed in subadult and young adult (Fig 1A), adults (Fig 1B) and aged females (Fig 1C). The youngest cycling female was 1.1 yr and the oldest was 13.2 yr. The mean duration of elevated FEM (estrus) was 4.4 ± 0.2 d (N = 57; range, 2–9 d), and was not affected by age group (*F*_2,19.5_ = 0.77; *P* = 0.48), parity (*F*_1,33.4_ = 0.78; *P* = 0.38) or prior contraception (*F*_1,35.6_ = 0.00; *P* = 0.99). Estrous cycle length averaged 17.5 ± 0.4 d (range, 8–30 d), and did not differ by age group (*F*_2,5.32_ = 0.11; *P* = 0.90), parity (*F*_1,6.16_ = 0.14; *P* = 0.72) or prior contraception (*F*_1,3.73_ = 1.59; *P* = 0.28). The one adult female (SB145) that exhibited periods of anestrous had been previously contracepted with MGA, but the implant was removed 2.6 yr (30.7 mo) before sample collections began. Although mean, baseline, and peak FEM in this lion were within the range of the other adult females, she exhibited three anestrous periods, averaging 78 ± 2.5 d (range, 73–81 d) over a 1.3-yr study period. Silent estrus was documented in 9 of 11 lions in the reproductive assessment portion where behaviors were collected, including SB406, SB247 (Fig 1A) and SB140 (Fig 1C). No behavioral estrus was recorded for SB406 and SB140, and SB247 rarely exhibited behavioral estrus over the 3 yr of sample collections.

**Fig 2:**
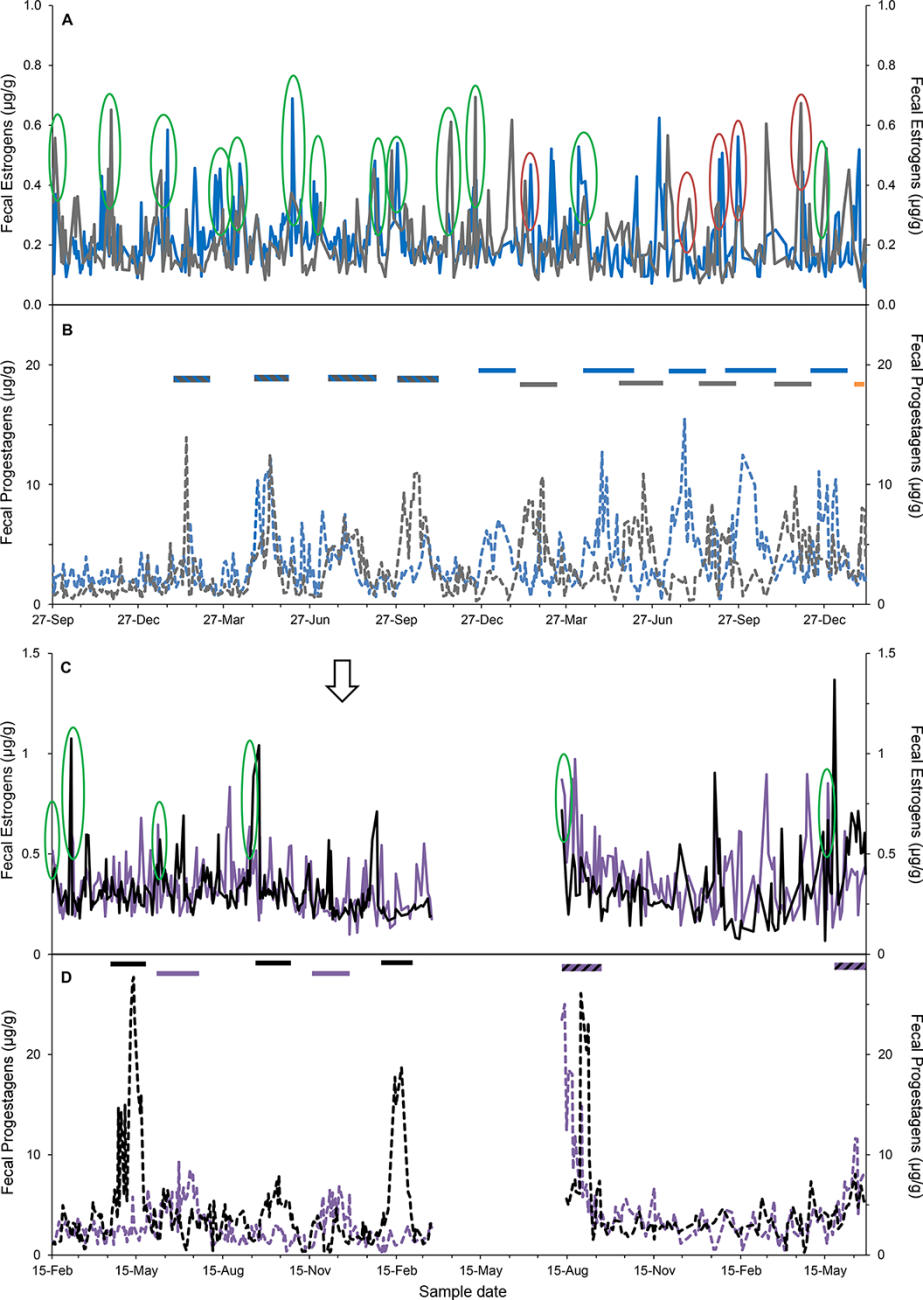
Synchronous estrous cycles and spontaneous ovulations in two pairs of female lions housed together for 2.4 yr. Panels A and B: SB246 (grey profile) and SB247 (blue profile); Panels C and D: SB170 (black profile) and SB172 (purple profile). Fecal estrogen metabolites (μg/g) are displayed in panels A and C with solid lines and fecal progestagens metabolites (μg/g) in panels B and D with dotted lines. Green circles indicate synchronized estrous cycles for the pair of females at each zoo and for SB246 and SB247, red circles show incidences of shared peak FEM while one female was in a luteal phase. Striped horizontal lines denote synchronized luteal phases caused by spontaneous ovulation events within a zoo, solid horizontal bars indicate luteal phases caused by spontaneous ovulations for each individual (not synchronized) and the orange horizontal bar represents the start of a pregnancy (SB246; the male was introduced to this female six weeks prior to the successful pregnancy). In panel C, the arrow signifies the arrival date of the male lion (SB215) to the zoo, but physical introductions between the females and the male were not conducted for more than 1.5 yr after his arrival.

Three facilities collected fecal samples simultaneously on paired females. Of those, synchronized ovarian events were observed in two pairs (Fig 2). Females SB246 and SB247, housed within visual, auditory and olfactory range of a male, but without direct contact, cycled synchronously 13 times over 2.4 yr (Fig 2A). Females SB170 and SB172 experienced synchronized estrous cycles four times without a male present, and on two occasions after a male arrived at the zoo with visual, auditory and olfactory, but not direct contact (Fig 2C).

### Pregnant and non-pregnant luteal phases

The mean duration of the NPLP was 46.0 ± 1.2 d (N = 25; range, 35–54 d) in 12 females; the youngest age that a NPLP was observed was 3.47 yr and the oldest age was 13.20 yr. Fifteen NPLPs were the result of spontaneous ovulations that occurred in females housed with another female, but no exposure to males (N = 4 individuals, e.g. Fig 2). One pair of spontaneously ovulating females, housed together, was within auditory, olfactory and visual range of a male, but did not have direct contact. These females also initially exhibited spontaneous ovulations after several synchronized estrous cycles (Fig 2A and 2B). However, during the second half of the sampling period, the cycles diverged and the females took turns cycling and ovulating. On several occasions, peak FEM were observed during NPLPs that corresponded with the other female’s estrous peak (red circles, Fig 2A and 2B). Two other females, housed together, spontaneously ovulated when housed both without and then within auditory, olfactory and visual range of a male. For the remaining 10 NPLPs, all females (N = 8) were housed with a male and likely were induced ovulations; seven of these occurred after documented breeding. There was no difference in NPLP length between spontaneous and induced ovulations (*F*_1,19_ = 0.28 *P* = 0.60), adult and aged groups (*F*_1,18.1_ = 0.11; *P* = 0.75), parous and nulliparous individuals (*F*_1,19_ = 0.50; *P* = 0.49) or in females previously contracepted versus untreated (*F*_1,19_ = 3.41; *P* = 0.08).

**Fig 3:**
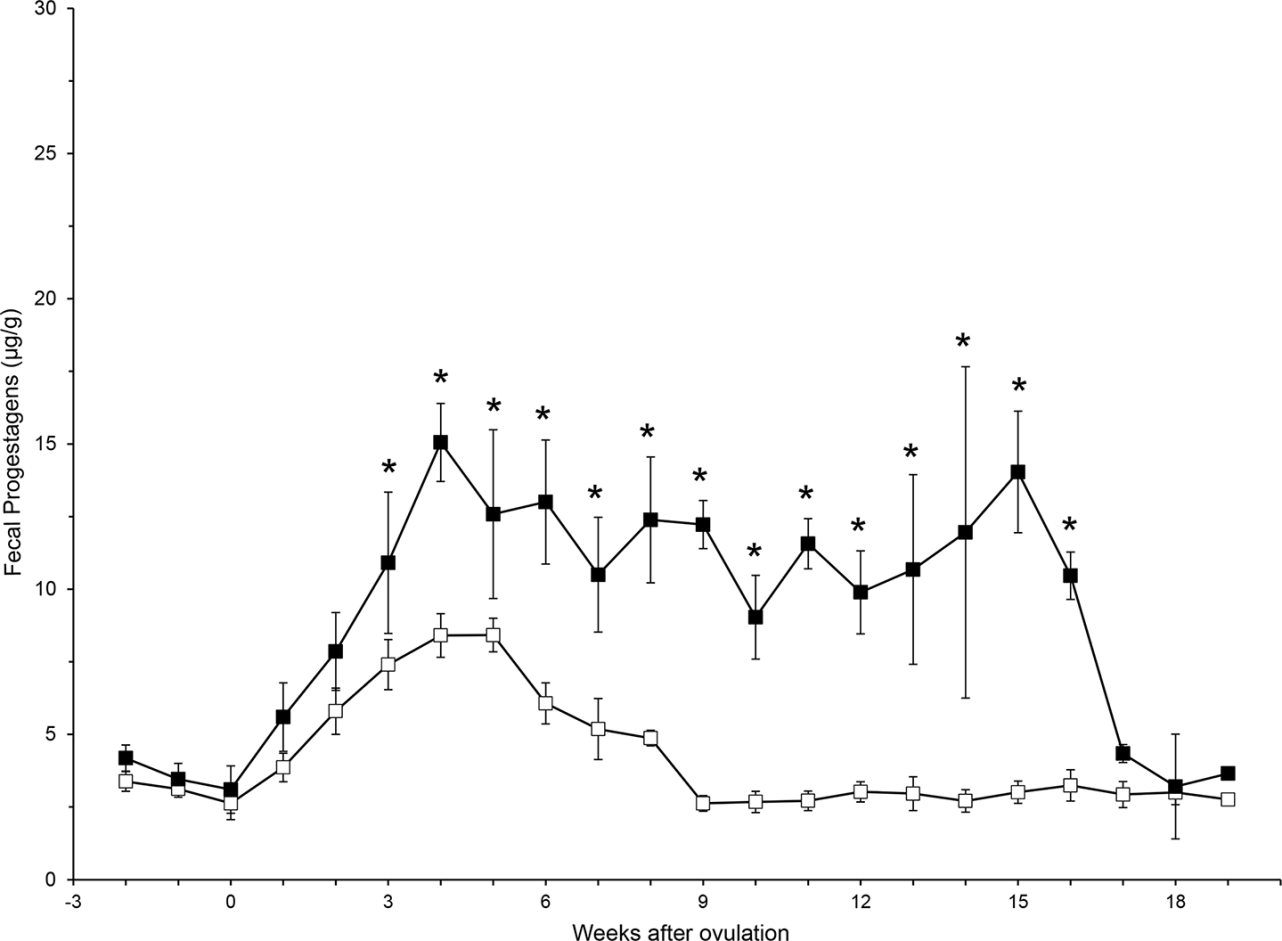
Weekly average (± SE) fecal progestagen metabolite concentration for pregnant (filled squares) and non-pregnant (open squares) luteal phases. Post-hoc Bonferroni correction analysis for significance between PLP and NPLP weekly average FPM concentrations are denoted by asterisks.

Eight complete pregnancies were observed in six individual lions, and averaged 109.5 ± 1.0 d in duration (range, 105–114 d). Within 3 wk of the start of a luteal phase, FPM concentrations were higher in PLP compared to NPLP (*F*_21,518_ = 5.19; *P* < 0.001) and remained elevated throughout the duration of gestation (Fig 3).

### Post-partum estrous cycles

**Fig 4:**
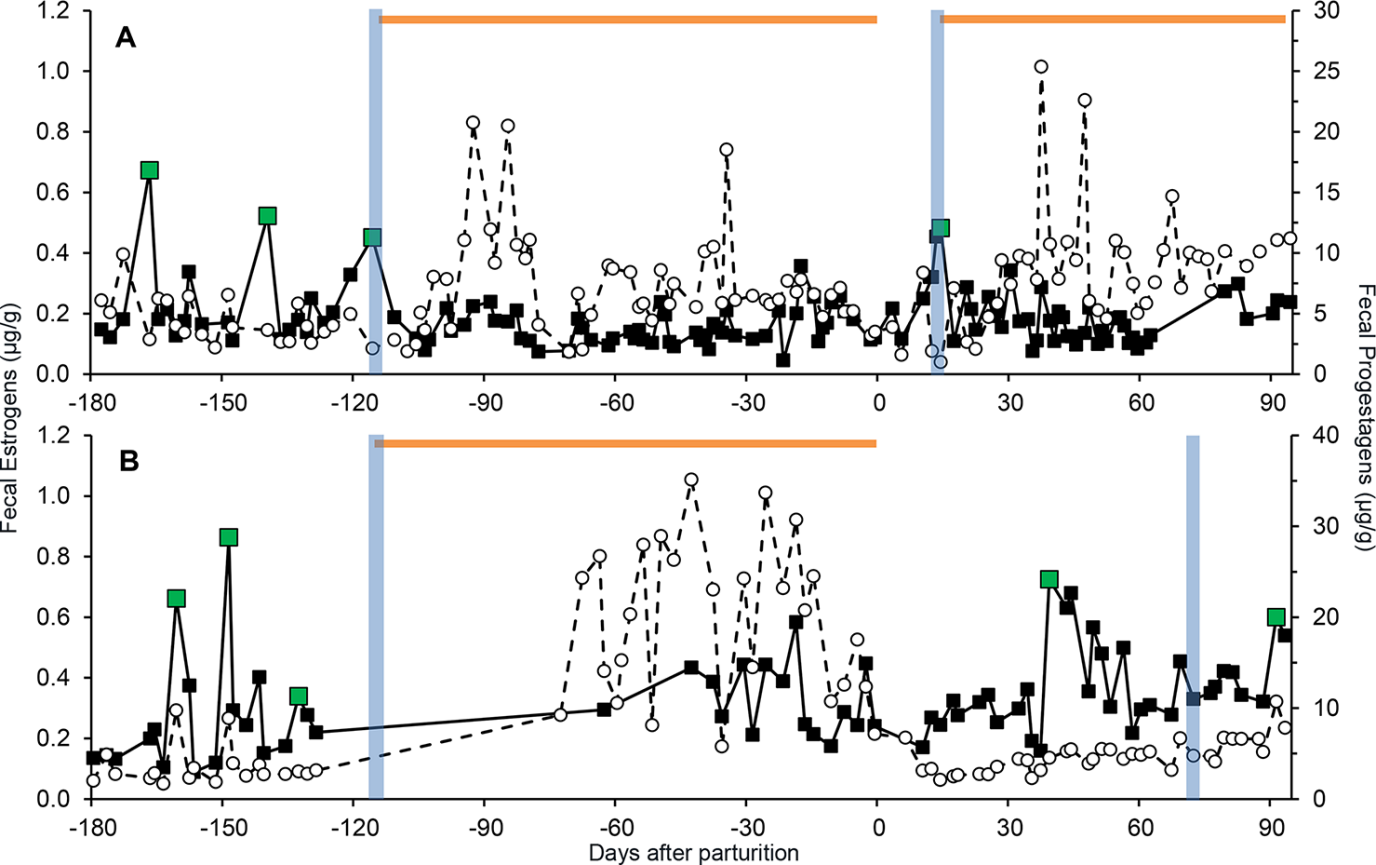
Longitudinal steroid hormone profiles (μg/g feces, FEM; filled squares, FPM: open circles, dashed line) showing the resumption of cycling post-partum in a female that lost her cub (SB246, Panel A) and a female with a surviving litter (SB341, Panel B). Green data points denote estrous peaks, vertical blue bars indicate breeding, the purple arrow signifies death of a cub and horizontal orange bars represent pregnancies.

In addition to eight complete PLPs, six partial pregnancies where the animal was already pregnant at the start of fecal sample collection were observed. In eight of the 14 pregnancies, sufficient sampling continued after parturition to observe post-partum estrous cycle resumption. Five of the eight litters did not survive more than a few days (accident, N = 1; illness, N = 2; stillborn, N = 2). Although not significant, on average, females that lost litters resumed cycling 16.4 ± 4 d (range, 11–22 d) post-partum sooner than those that nursed cubs (31.7 ± 5.9 d; range, 20–39 d (*F*_1,3.9_ = 6.86; *P* = 0.07). Females without surviving cubs bred with a male sooner than females with live cubs, averaging 23.8 ± 12 d (range, 12–40 d) after birth compared to 63 ± 3 d (range, 61–65 d) (*F*_1,5_ = 20.88; *P* = 0.01). Female SB246 lost her cub to illness at 3 d post-partum; she exhibited peak FEM 12 d after giving birth and was observed breeding at 13 d post-partum, resulting in a PLP (Fig 4A). While caring for and nursing 3 cubs, SB341 was allowed access to a male for a few hours a day. She cycled 39 d after parturition without breeding, but bred at her second cycle 65 d post-partum (Fig 4B).

### Effect of contraception

**Fig 5:**
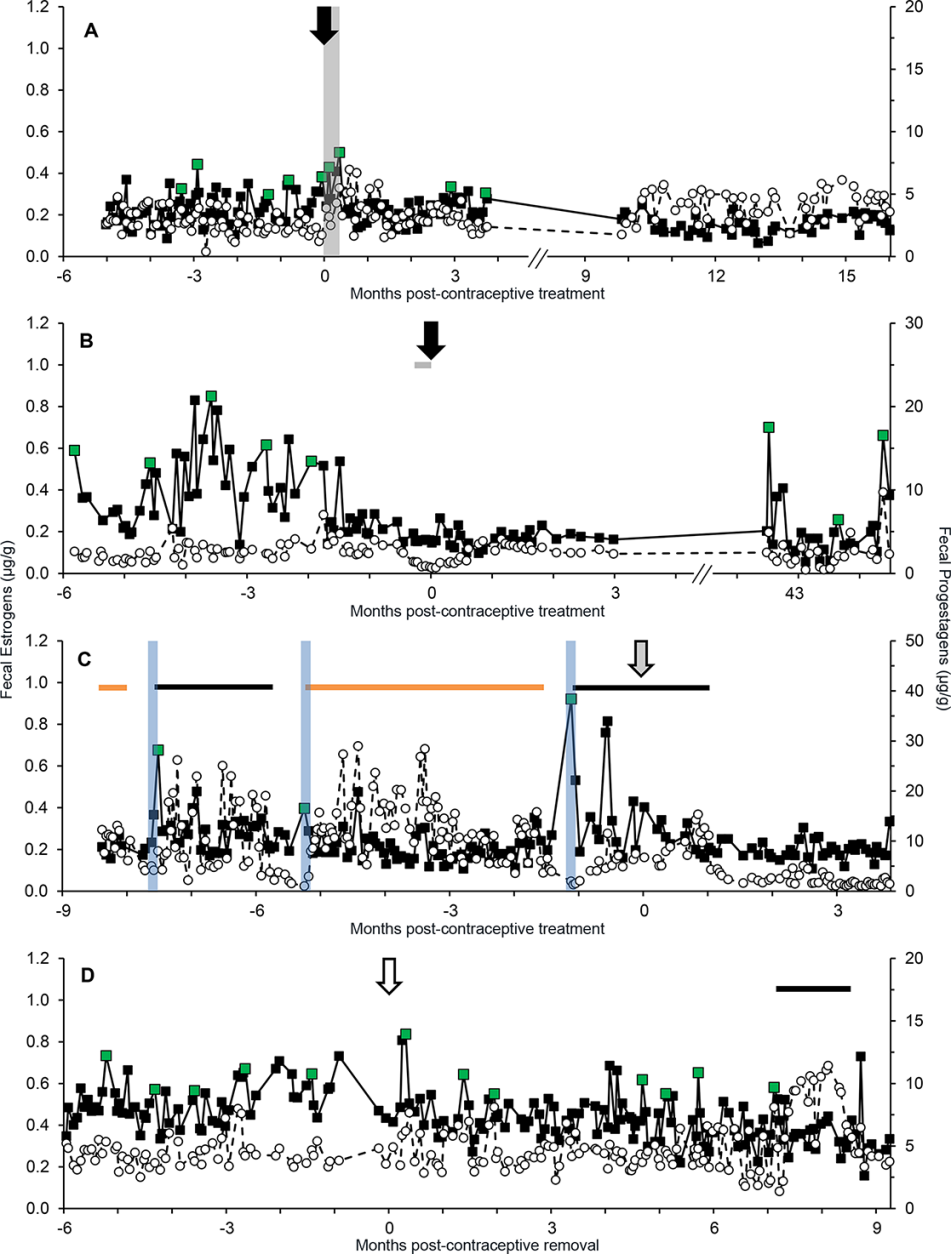
Reproductive steroid hormone profiles (μg/g feces, FEM: filled squares, FPM: open circles, dashed line) before and after Suprelorin or MGA treatment and before and after MGA removal. Suprelorin treatment indicated with black arrows in panel A (SB244) and panel B (SB341), MGA treatment represented with a grey arrow in panel C (SB124) and MGA removal denoted with an open arrow in panel D (SB224). Green data points indicate estrous peaks, vertical blue bars correspond with breeding, black horizontal bars represent non-pregnant luteal phases and orange horizontal bars signify pregnancies. In panel A, the vertical grey bar shows a 9 d contraception-induced estrous event. In panel B, the horizontal grey bar denotes a 14 d oral megestrol acetate (MA) treatment prior to Suprelorin placement.

Eleven females were contracepted with Suprelorin or MGA implants or with a Depot Lupron injection during at least part of the study period. Eight females were implanted with Suprelorin, three of which were monitored both before and after treatment (Table 2). Longitudinal profiles of two females before and after Suprelorin treatment are shown in Fig 5. The female in Fig 5A (SB244) was monitored for 5 mo before being contracepted with Suprelorin, for 3.7 mo post-implant, and then again 9.9–16.3 mo after initial contraception. Overall (*F*_1,166_ = 18.13; *P* < 0.001) and baseline (*F*_2,149_ = 21.10; *P* < 0.001) mean FEM production differed among the sampling periods. While there were no differences between pre-treatment and the first 3.7 mo after contraception (mean, *P* = 0.43; baseline, *P* = 0.08), lower mean and baseline FEM were observed during the time period 9.9–16.3 mo post-treatment when compared to pre-treatment (mean, *P* < 0.001; baseline, *P* < 0.001), and the immediate post-treatment sampling period (mean, *P* < 0.001; baseline, *P* < 0.001) (Table 2). By contrast, SB341 (who received 2 weeks of megestrol acetate surrounding the day of Suprelorin implant placement, to prevent the initial GnRH agonist stimulation phase) showed an immediate decrease in mean and baseline FEM after implant placement through the remaining 3 mo of sample collection (mean, *F*_2,132_ = 22.35, *P* < 0.001; baseline, *F*_2,106_ = 47.81, *P* < 0.001) (Fig 5B). When sampling resumed 41.8 mo post-treatment, she was cycling and her mean and baseline FEM had increased, although not to pre-contraception concentrations (*P* < 0.001) (Table 2; Fig 5B). Not all females contracepted with Suprelorin exhibited a complete suppression in ovarian activity as evidenced by similar FEM concentrations pre- and post-implant (e.g., SB109; *F*_1,68_ = 0.01; *P* = 0.94) (Table 2). Also, a post-contraception rise in FEM concentrations for 9 d was observed in SB244 (who was not given megestrol acetate to prevent the stimulation phase), but not SB341.

**Table 2:** Overall and baseline mean (± SE) concentrations of fecal estrogens before and after Suprelorin and MGA contraception treatments.

				Fecal Estrogens (μg/g)		Fecal Progestagens (μg/g)	
Type	SB	Age at treatment	Months after treatment*	Overall	Baseline	Overall	Baseline
Suprelorin	109^+^	13.6	-3.5–0	0.23 ± 0.01^a^	0.22 ± 0.01^a^	5.16 ± 0.34^a^	3.27 ± 0.20^a^
			0–2.4	0.22 ± 0.02^a^	0.22 ± 0.02^a^	2.85 ± 0.51^b^	2.28 ± 0.40^b^
	244	2.0	-5–0	0.22 ± 0.01^a^	0.21 ± 0.004^a^	2.77 ± 0.11^a^	2.42 ± 0.09^a^
			0–3.7	0.24 ± 0.01^a^	0.22 ± 0.01^a^	3.37 ± 0.16^b^	2.95 ± 0.10^b^
			9.9–16.3	0.16 ± 0.01^b^	0.16 ± 0.01^b^	4.08 ± 0.18^c^	4.02 ± 0.18^c^
	341^ɣ^	2.0	-7.1–0	0.36 ± 0.02^a^	0.22 ± 0.01^a^	2.69 ± 0.11^a^	2.38 ± 0.07^a^
			0–3	0.17 ± 0.03^b^	0.16 ± 0.01^b^	2.51 ± 0.19^b^	2.51 ± 0.19^a^
			41.8–51.8	0.24 ± 0.03^b^	0.15 ± 0.01^b^	2.78 ± 0.32^c^	2.13 ± 0.16^b^
MGA	124^θ^	8.9	-8.4–0	0.26 ± 0.01^a^	0.21 ± 0.01^a^	4.44 ± 0.40^a^	3.72 ± 0.30^a^
			0–4.5	0.21 ± 0.01^b^	0.19 ± 0.01^b^	2.04 ± 0.11^b^	1.54 ± 0.05^b^
	233	7.4	-4.6–0	0.29 ± 0.02^a^	0.27 ± 0.01^a^	2.38 ± 0.10^a^	2.30 ± 0.04^a^
			0–1.7	0.26 ± 0.01^a^	0.26 ± 0.01^a^	2.37 ± 0.07^a^	2.30 ± 0.07^a^
	224	~7.8	24–30	0.52 ± 0.01^a^	0.51 ± 0.01^a^	5.05 ± 0.14^a^	4.29 ± 0.09^a^
			30.1–39^	0.43 ± 0.01^b^	0.39 ± 0.01^b^	4.41 ± 0.14^b^	3.80 ± 0.09^b^

^a,b,c^ Within each animal, hormones are compared by column and different subscripts indicate significant difference before and after treatment (*P* < 0.05).

*Months post-contraceptive treatment

^^^Months after MGA removal

^+^SB109 was previously treated with DMPA– 10/27/1999 and Suprelorin– 12/1/1999: 2 x 6mg (older formulation). SB109 gave birth 5/23/2002, a reversal 2.5 yr after treatment with the old Suprelorin formulation.

^ɣ^SB341 was given oral MA for 14 days around the day of Suprelorin implant placement and the data from those samples are not included in the average hormone values.

^θ^SB124 was previously treated with MGA—7/21/1998, 1/21/1999 and 12/17/2003. SB124 gave birth 6/14/2002 and 12/5/2004, reversals of the previous two MGA treatments.

Two females (SB124, SB233) were monitored before and during treatment with MGA (Table 2; e.g., SB124, Fig 5C) and one (SB224) during MGA (collections began ~2 yr after implant was placed) and after implant removal (Fig 5D). In SB124, mean and baseline FEM concentrations decreased (*F*_1,176_ = 6.47; *P* = 0.01 and *F*_1,151_ = 5.01; *P* = 0.03, respectively), whereas no changes were observed for SB233 in mean (*F*_1,64_ = 1.50; *P* = 0.23) or baseline (*F*_1,63_ = 0.83; *P* = 0.37) FEM. No cycles were observed for the remainder of sample collections for SB233 (55 d), and in SB124 (137 d) only one small peak in FEM occurred. Fig 4C demonstrates SB124 as a cycling adult female, with PLPs and NPLPs until she was contracepted with MGA. The length of the second NPLP (48 d) was not affected by the contraception treatment as it is within the range of NPLP lengths observed during this study. In SB224, there were presumed waves of follicular development based on FEM while the contraceptive implant was in place. After removal, both mean and baseline FEM increased (mean: *F*_1,216_ = 52.70; *P* < 0.001; baseline: *F*_1,201_ = 90.97; *P* < 0.001) (Table 2, Fig 5D)**.** SB224 exhibited a wave of follicular development 7 d after the contraceptive implant was removed and experienced a NPLP 7.1 mo later.

**Fig 6:**
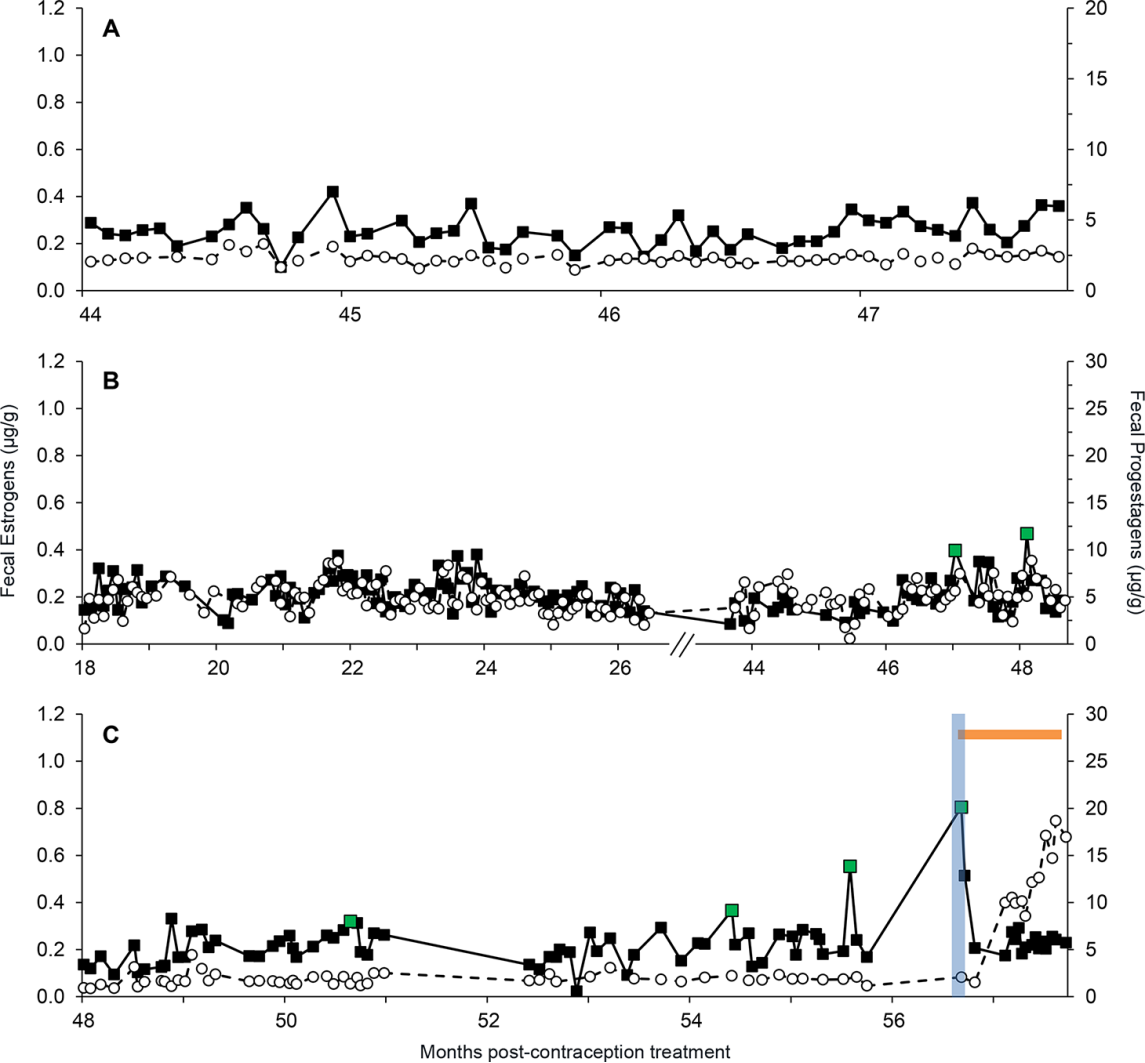
Fecal steroid hormone profiles (μg/g, FEM: filled squares, FPM: open circles) for three females contracepted with Suprelorin. Panel A (SB200), Panel B (SB283), Panel C (SB218). Baseline and threshold estrogen concentrations are denoted by dashed and dotted lines, respectively. Green data points, a blue vertical bar and an orange horizontal bar indicate estrous peaks, breeding and pregnancy, respectively.

The average time from Suprelorin treatment to resumption of cycling was 48 mo (N = 3, range, 42.2–54.4 mo) (S1 Table). The average duration of efficacy in females that had not reversed by the end of the study period was 5.2 yr (62.4 mo) (N = 12; range, 41.3–78.5 mo). Female SB283 (Fig 6A) did not cycle until 47.4 mo after treatment, and the peak FEM concentrations were relatively low at those estrous cycles. Female SB200 (Fig 6B) was treated with Suprelorin annually for 3 yr prior to the last dose, for a total of 107.5 mo of contraception treatment. The mean length of time between the last administration of Suprelorin and those that did fully reverse by giving birth was 4.3 yr (51.7 mo) (N = 6; range, 31.4–66.8 mo). A resumption of cycling was observed during the study period in SB218 at 50.6 mo after contraceptive removal, and she conceived at 56.7 mo (Fig 6C), whereas SB341 had returned to cycling when collections resumed 42 mo after contraceptive removal, and she mated and conceived at 45.5 mo, giving birth 49.1 mo post-contraceptive treatment.

**Fig 7:**
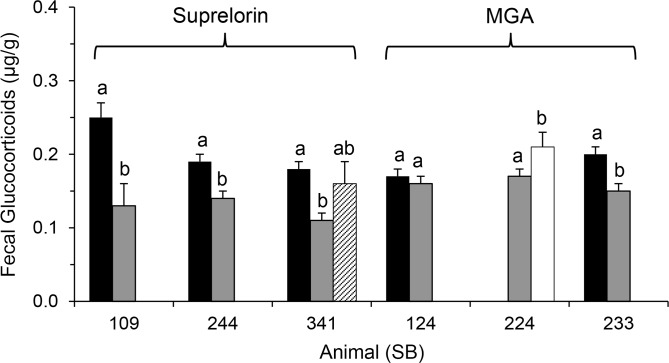
Comparison of mean FGM concentrations (μg/g ± SE) before birth control implant placement (black bars), on birth control treatment (grey bars), after expiration of GnRH-agonist (striped bar) and after removal of progestin (white bar) contraception implants. Within individual, different letters denotes significance (*P* < 0.05).

Mean and baseline FGM concentration decreased after treatment with Suprelorin; SB109 (*F*_1,68_ = 11.43; *P* = 0.001); SB244 (*F*_2,180_ = 4.26; *P* = 0.02); and SB341 (*F*_1,94_ = 19.50; *P* < 0.001) (Fig 7). One of two individuals implanted with MGA also experienced a reduction in mean and baseline FGM; SB124 (*F*_1,280_ = 0.81; *P* = 0.37) and SB233 (*F*_1,77_ = 17.78; *P* < 0.001), and when the MGA implant was removed from SB224, both mean and baseline FGM increased (*F*_1,203_ = 4.54; *P* = 0.03) (Fig 7).

### Body weights

**Fig 8:**
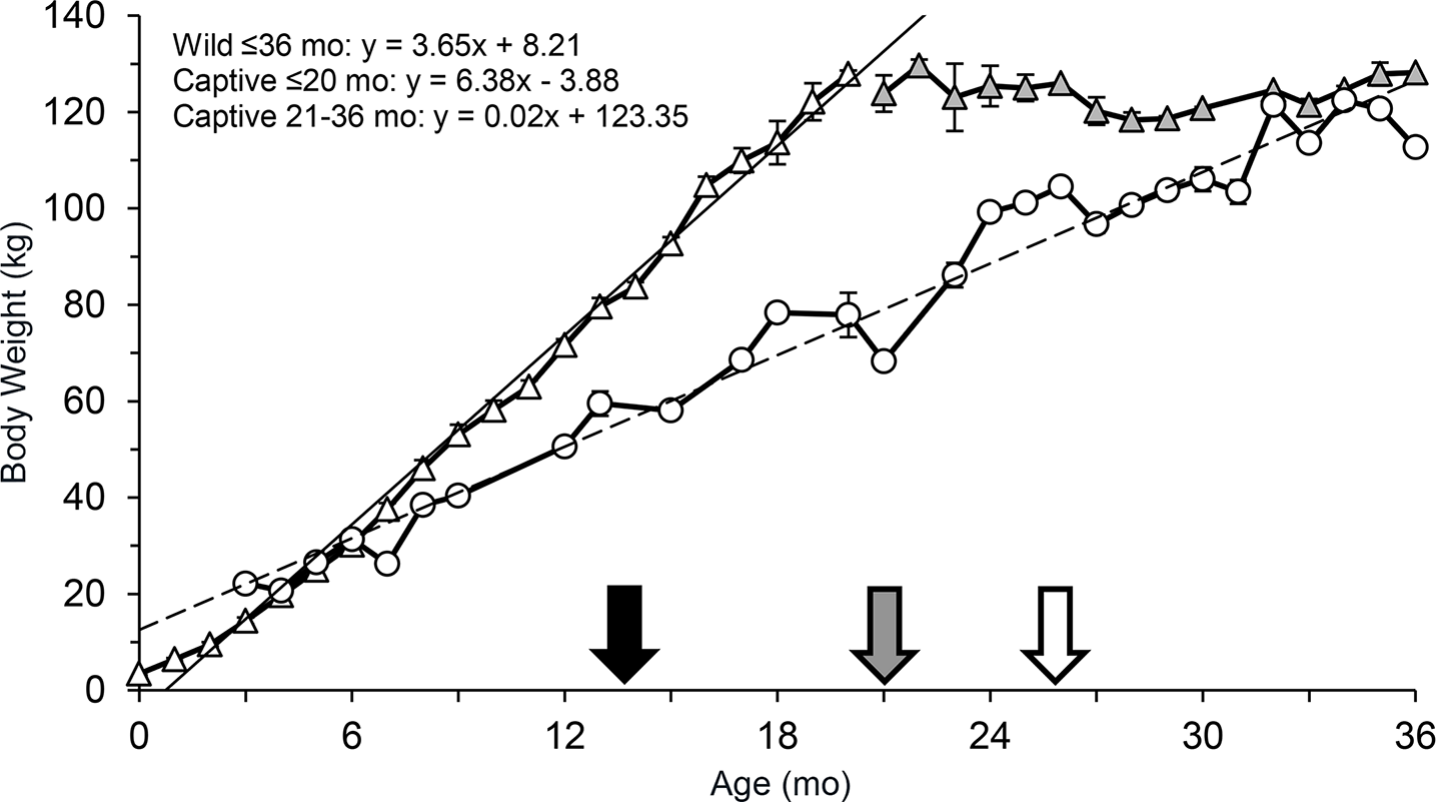
Monthly average (± SE) body weights (kg) of captive (triangles) and wild (circles, adapted from Smuts et al. (1980)) female lions through 3 yr (36 mo). White triangles denote months when captive females were actively gaining weight and grey triangles represent months where the average body weight was similar to adult weights. Trend lines are solid for captive lions and dashed for wild lions. The black arrow indicates the average age, 13 mo (1.06 yr), and body weight, 80 kg at which captive females (n = 4) showed waves of follicular development. The grey arrow approximates when wild lions should begin cycling based on the age, 21 mo (1.75 yr), at which they reach ~80 kg and the open arrow is the estimated age (26 mo, 2.2 yr) and body weight (96 kg) when females reach puberty in the wild using behavioral observations (Smuts, 1980).

There were differences in average monthly body weights between captive and wild cubs (*F*_1,237_ = 177.80 *P* < 0.001), although the direction differed by age categories. The estimated average monthly weights for wild cubs aged 0–2 mo was higher than their captive born counterparts (Month 0, *P* < 0.001; Month 1, *P* < 0.001; Month 2, *P* = 0.004; Fig 8). From 3–5 mo, there was no difference (Month 3, *P* = 0.07; Month 4, *P* = 0.55; Month 5, *P* = 0.37), but then from 6 through 20 mo, captive-born cubs weighed more than wild cubs (*P* = 0.01 at 6 mo; *P* < 0.001 for 7–20 mo). Regression analysis estimated that captive lions gained 6.37 kg/mo [212.3 g average daily gain (ADG)], while wild lions gained 3.07 kg/mo (102.3 g ADG). Weight comparison between wild and captive individuals ended at 20 mo because the captive lions had reached an average adult weight of ~125 kg. Wild lions continued to gain weight through the end of data collection at 36 months.

## Discussion

This is the first comprehensive analysis of longitudinal reproductive and gonadal steroid hormone patterns in female African lions, and highlights the effects of age, adrenal status and contraceptives on reproductive activity. Methods were developed for assessing basic endocrine function in lions noninvasively, including determining the excretion of relevant metabolites in feces and validating immunoassays. Results suggest that captive lions reach puberty at an earlier age than their wild counterparts, which correlated with different rates of body weight gain over the first 2 years of life. Mean FPM, but not FEM and FGM, concentrations varied by age, and the lengths reproductive parameters such as estrous cycle and PLP were similar to previously published data. The lack of anestrous observed within the reproductive assessments (5%) indicates that ovarian acyclicity was not a cause of poor reproductive output after the breeding moratorium was lifted, but may be a consequence of management decisions relating to housing, diet or animal personality [47]. Additionally, we found that contraception with Suprelorin implants delayed the return to cyclicity for an extended duration and that both progestin and GnRH agonist contraception types elicited a decrease in FGM production during treatment.

Based on HPLC analysis, unconjugated estradiol-17β was the major estrogen metabolite found in fecal extracts, which agrees with findings in other felid species [41]. Moreover, estradiol-17β was positively correlated with reproductive behaviors in female lions and determined to be a good method to monitor waves of follicular activity [48]. Unlike estrogens, progesterone is more heavily metabolized in felids and excreted as both polar and nonpolar metabolites, but not as native steroid [41,49], which is similar to HPLC results in lions. By contrast, the cortisol EIA determined that native steroid constitutes the majority of immunoactivity in HPLC fractions, which differs from findings in other felids, including domestic cats (*Felis catus*) [50], cheetahs (*Acinonyx jubatus*) and clouded leopards (*Neofelis nebulosa*) [51].

One of the major findings, based on evaluation of FEM, was the early age that captive female lions experience puberty. Waves of estrogenic activity, indicative of follicular development, were observed in lions as young as 1.1 yr of age, and mean and baseline FEM were comparable to older individuals. Wild females were believed to become sexually mature at around 26 mo of age, based on observations of estrus behaviors such as lordosis and rolling, and have an average age of first conception of 3.5–4.5 yr [32,52]. This is later than what was noted in our zoo lions based on endocrine activity. The cyclicity findings agree with growth data showing captive lion cubs appear to develop faster than wild counterparts. In most mammals, puberty is dependent on an adequate body weight [53–57] and fat reserves [58], presumably related to good nutrition. For example, age at puberty in heifers is inversely related to body weight [56], and captive-raised black-tailed deer fawns (*Odocoileus hemionus columbianus*) bred at 5–6 mo, whereas wild counterparts did not conceive until the following breeding season, at > 1 yr [59]. By 20 mo of age, captive lions in this study reached their adult weight, whereas wild lions continued to grow through 36 mo of age [32]. Captive female lions began cycling at 1.1 yr of age at an average weight of ~80 kg, whereas wild counterparts reached a similar weight at 1.75 yr. If body weight is a good predictor of puberty onset in this species, then it is likely wild females reach puberty at an earlier age than is currently believed based on estrous behaviors, especially given our finding that silent estrus is common in lions. The studbook lists three females that conceived before 2 yr of age and all litters survived [27]. It may be prudent to study the impact of rapid growth on young lions as well as future reproductive capability and overall health, although an accelerated growth rate did not impair reproductive function or longevity in dairy cattle [56]. One study of longevity in captive lions found that age at first reproduction and number of offspring also did not appear to impact lifespan [60].

The finding that FEM patterns were comparable in female lions across reproductive age groups indicates that ovarian function remains relatively constant throughout a female’s lifespan once cycling has initiated [61]. Similarly, average FEM concentrations did not differ with age in cheetahs (2–15 yr) [62], although in Arabian leopards (*Panthera pardus nimr*), baseline FEM were highest in young females (< 2 yr of age) and lowest in older individuals (> 2–20 yr) [63]. Higher overall mean FPM in lions after the age of 3 yr, which then remained constant for the remainder of adulthood, may be related to the onset of ovulations, as none of the animals in the sub-adult group exhibited NPLPs. In contrast, FPM did not vary by age group in cheetahs [62]. Whether lions experience a true reproductive senescence is not clear. Smuts et al. [51] reported decreased ovarian follicle number in aged (11–14 yr) wild lions and Packer et al. [63] observed age-related reductions in fecundity. Yet wild lions are capable of reproducing up to the time of death (~16 yr of age) [19,64,65]. According to the African lion studbook, lions as old as 14.9 yr of age have reproduced. But because pregnancy complications are more prevalent in very young and old animals [66], older lions often are not given breeding recommendations, thus potentially masking any age-related decreases in reproduction.

Estrous cycle lengths of 19 d (N = 2; range, 18–20 d) [31] and 20.4 ± 1.7 d [48] have been reported for lions, which is similar to our findings and those of behavioral observations of wild lions [65]. Cycle length also varied considerably within and between individuals, as observed in other felid species, including the cheetah (13.6 d; range, 5–30 d [34]), puma (17–25 d [67]), clouded leopard (24 d; range, 14–43 d [68]), tiger (*Panthera tigris* ssp; 17.96 d; range, 6–40 d [69]), and *Leopardus* genus: margay (*L*. *wiedii*; 17.6 d; range, 11–25 d), ocelot (*L*. *pardalis*; 18.4 d; range, 7–31 d) and tigrina (*L*. *tigrinus*; 16.7 d; range, 11–27 [44]). For solitary felids, variable estrous cycle lengths may increase the probability of being in estrus when encountering a male. Lions are the only social lion species, and estrous synchrony has been observed in the wild, especially after male take-overs [27]. However, this behavior does not occur in captive prides, so variable estrous cycle lengths were not unexpected in our study. Peaks in FEM can be indicative of estrus in felids [48,70]; however, because results are delayed by the processing methods, real-time diagnosis of estrus with fecal hormone metabolites is unrealistic. Daily accounts of behaviors are still necessary to time breeding introductions, particularly with singly-housed animals. The duration of estrus in our study ranged from 2–9 days. Other felids experience variable lengths of estrus as well, including the clouded leopard (6 d; range, 1–10 d) [68], cheetah (2–6 d), lynx (< 7 d) [18] and *Leopardus* spp (1–6 d) [44].

Not all FEM peaks were associated with behavioral estrus, however, and thus represented silent estrus, which has been noted previously in lions [71], as well as other felids, including the cheetah [72], domestic cat [73] and Arabian leopard [63]. Silent estrus was observed in 73% of the females in this study where behaviors were available (N = 11, S1 Table). No estrous behaviors were observed in the six sub-adult females, despite the occurrence of regular increases in FEM. It is possible that a threshold of estrogen production is needed to stimulate overt behaviors in lions, but that is unlikely, as there was high variation in mean and baseline concentrations within and among all females. Another possibility is that follicle growth rate in some females or during some cycles may be slower; a study monitoring follicular maturation via ultrasound in water buffalos (*Bubalus bubalis*) found a link between slow growing follicles and silent estrus [74]. In lions, males are the submissive breeding partner [75] and low or slow estrogen production could preclude a female’s initiative for breeding, reducing reproductive success between a mating pair. The majority of females experiencing silent estrus were housed with males; only SB244, SB247 and SB341 were not housed with a male while cycling, and SB247 continued this pattern even after being housed with the male. Thus, the expression of overt estrous behaviors was not influenced by the presence of a male.

The NPLP length of 35–54 d was within the range of that reported previously in lions [48,71,76] and other felids, including the snow leopard (*Panthera uncia*; 42–53 d) [77], Arabian leopard (39 d) [63], caracal (*Caracal caracal*; 47–48 d) [48] and cheetah (38–59 d) [34]. The finding that 60% of NPLPs occurred in the absence of a male indicates that lions can ovulate spontaneously, as was observed in other studies involving both group and singly housed lions [31,48,71].

The analysis of fecal hormone metabolite patterns of two pairs of group-housed sisters without direct male contact presented evidence of estrous cycle synchrony, which has been noted in wild lions [28,29,65]. Synchrony in estrous cycles [28,65] and births [28,29,65] both have been observed within females of the same pride, but not across prides in the same region [28]. Synchronous estrous cycles are thought to increase offspring survival because females can share the responsibility of caring for young and more successfully protect their young from infanticide by a new pride male [28,29]. One theory for the source of synchrony is exposure to pheromones of cycling females in close proximity–a mechanism comparable to the dormitory effect proposed in women (*Homo sapiens*) [78] and observed in kenneled domestic dogs (*Canis lupus familiaris*) [79]. But while synchronized estrus and breeding have been observed in wild lions, this is the first observation of ovarian synchrony involving successive NPLPs.

The average PLP length observed in the current study was in agreement with earlier findings that gestation lasted 110 d (range, 100–114) [75], 102 d ± 3.02 [30] and 115 d (range, 111–119) [80]. In this study, overall mean FPM concentrations during gestation were higher than mean NPLP levels starting 3 wk after ovulation, which contradict an earlier report wherein no difference in FPM concentrations was observed between PLP (n = 1) and NPLP (n = 3) female lions [48]. This discrepancy may be attributed to the small sample size utilized by Graham et al. [48]. Similarly, domestic cats and Arabian leopards excrete more progestagens during the PLP than the NPLP [63,81]. Still, the variation in hormone concentration within and among individuals prevents accurate pregnancy diagnosis prior to ~60 d after breeding by which time elevated FPM in a NPLP would return to nadir.

Our findings agree with earlier studies of wild lions where females that lost their litters typically resume cycling soon thereafter [28–30]. In our study, five litters were lost and females resumed cycling within 11 to 22 d. Cycling in domestic cats has been shown to resume within 7 d of litter removal, 1–2 d after parturition [82], compared to ~4 wk for nursing females [83], and within 30 d in a clouded leopard that rejected her cub, compared to several months in lactating females [68]. However, in contrast to most reports that lions with living litters do not cycle for at least 18 mo after giving birth [29,65], lactating lions in this study experienced estrous cycles within weeks of parturition based on waves of FEM excretion, and in one case, breeding, which has been observed in lactating wild lions [79,84]. The lion studbook provides evidence of at least one example of a dam successfully rearing two litters born < 1 yr apart [27].

This study provides some of the first documentation on the impact of contraceptive usage on steroid hormone production and post-treatment reproductive capabilities in African lions. As Suprelorin is designed to fully suppress FSH and LH production, a decrease in mean and baseline FEM and FPM was expected [37]; FPM decreased in all three females monitored before and after treatment, and FEM was lower in two of three individuals. It is possible that lower FEM would have been observed in SB109 if the duration of sampling was longer because the FEM did not immediately decrease in SB244 either. It is not clear why MGA also caused a reduction in FEM and FPM in one of the two individuals treated. Synthetic progestins generally do not fully suppress FSH and LH production and waves of follicular development and even ovulation have been observed in cattle [85,86]. Low and medium dosages of altrenogest, an oral synthetic progestin, in domestic cats did not cause a change in estrogen or progestagen production, whereas high concentrations of the drug actually increased ovarian steroid concentrations [87]. Thus, the effect of synthetic progestins on felid ovarian function has not been consistent.

While lions treated with MGA have cycled and bred soon after implant removal (28 ± 4 d; N = 3) [88], sustained treatment (> 2 yr) with MGA is associated with reproductive organ pathologies in felids [39]. The AZA Wildlife Contraception Center (WCC) used to recommend using MGA for a maximum of 4 yr and removing the implants after 2 yr to allow for a pregnancy before retreatment [89,90]. But starting in ~2005, Suprelorin implants began to be used in lieu of MGA because no adverse effects had been linked to Suprelorin use in felids [91]. However, Suprelorin implants are not easily removed, and we now know the duration of efficacy is wide-ranging [92]. Studies of Suprelorin treatment in domestic cats and Wistar rats (*Rattus norvegicus*) found many females failed to resume normal ovarian cyclicity after the duration of expected efficiency [91,93–95]. Additionally, after a year of Suprelorin treatment, the ovaries and uterine horns in rats were smaller, had fewer pre-antral follicles, and overall ovarian and uterine volumes were lower compared to control individuals [96]. The most recent data reported to the WCC indicate that the reversal rate (i.e., number of individuals that have so far produced cubs out of the number recommended to breed) of female lions is about 40% (M. Agnew, personal communication). We confirmed the extended efficacy duration of Suprelorin in several females through sampling > 40 mo post-treatment. The duration of Suprelorin’s impact on the HPG axis was longer [4.0 yr (48 mo)] in the lions in the present study compared to 33.8 mo in previous studies of wild and captive lions in South Africa [97], but both studies showed that the effect was considerably longer than the 1-yr predicted efficacy. It is now clear that the stated 6- or 12-mo periods of Suprelorin efficacy are minimal, and most individuals should be expected to be suppressed beyond that time. Additionally, the age an animal is treated and the dosage of Suprelorin utilized likely impacts the duration of efficacy. Given the unpredictability of time to reversal, the current recommendation of WCC is to place implants in a location that will facilitate removal when breeding is recommended (for instructions on placement and removal: http://www.stlzoo.org/animals/scienceresearch/contraceptioncenter/). In domestic cats, estrus and ovulation were induced when stimulated with eCG/hCG 10 d after Suprelorin implant removal [98] so it is possible that a similar course of treatment could reverse the prolonged duration of efficacy in lions. Additionally, while a study of Suprelorin-treated queens showed an average duration of efficacy of 22.7 ± 2.0 mo, 88% of female domestic cats mated immediately upon resumption of cycling conceived and carried litters to term [95], indicating that Suprelorin is a reversible contraceptive treatment in cats. As a result of these findings, the WCC has started recommending MGA contraception again with strict directions for its safe usage to avoid negative side effects: do not treat an animal until after her first successful pregnancy, allow a pregnancy between treatments, and limit each individual to only two MGA treatments. This change will provide more opportunities to explore how synthetic progestins affect hormone production in lions.

An interesting finding was that almost all lions contracepted during this study showed a significant decrease in FGM excretion, whether treated with progestins or GnRH agonist. In an earlier study in lions, only a high dose of MPA but not MGA caused significantly lower serum cortisol concentrations compared to controls [99]. Furthermore, in that study, the dose of MGA associated with elevated cortisol was 10 times higher than the contraceptive dose now recommended for lions, and the dose of MPA was two to four times higher than used in lions (AZA WCC Contraceptive Database). Adrenocortical suppression has been reported in domestic cats and humans treated with high doses of another synthetic progestin, megestrol acetate (MA) [100–102], and in dogs contracepted with a high dose of MPA [103,104], albeit only transiently (e.g., 4 wk) [103]. Although these studies used much higher doses than are recommended for zoo animal contraception, they do emphasize the need for caution in following the recommendations and accurately calculating the dose per kg body weight for each individual.

Little information is available on the effect of GnRH agonists on HPA function. In a study of common marmosets (*Callithrix jacchus*), treatment with the GnRH agonist leuprolide resulted in lower cortisol in intact but not in ovariectomized females. Those results suggest that the effect may be secondary to changes in ovarian hormones associated with ovulation rather than with basic adrenal function [105]. Two possible mechanisms for higher cortisol in cycling females are 1) physiological or physical changes caused by estrogen and/or other ovarian hormones (e.g., estrogen-stimulated increases in activity in some species) or 2) social stress during estrus related to interactions with courting males or competing females [106,107]. The results of this study of lions is important in emphasizing the importance of endocrine monitoring of both natural cycles and of contraceptive use. It is also important to always report any potentially negative effects of contraceptives to the WCC for follow up (www.stlzoo.org/contraception).

In summary, this study represents the most extensive dataset on the normal reproductive biology of female lions and shows how age and contraception affects steroid hormone production. The period of low fecundity observed between 1999 and 2004 now appears to have been reversed, and in 2013, the SSP set a goal of producing 30 cubs with 45 being born (H Colahan, personal communication). Ironically, this is leading us back to the problem we had before the moratorium, as there once again is a need to control population growth. Many zoo populations fluctuate in boom–bust cycles, which can create challenges when trying to maintain gene diversity and a stable population size. As such, an institution’s breeding recommendations are made as and when they are requested by the genetic needs of the population, and can be sporadic over time. Additionally, since this study was undertaken, a multi-institutional survey of lion management and husbandry practices concluded that housing and animal temperament impacted an individuals’ reproductive success. Specifically, housing lions in larger pride sizes (more than one male and one female) and bold personality types that were not shy about approaching keepers for training were associated with improved reproductive output [47]. It is possible that a change in management protocols was part of the cause for poor reproduction. Also, knowledge loss regarding the successful breeding of lions in captivity due to experienced staff retiring or moving to other jobs during the moratorium could have been a contributing factor in the decreased reproductive success. Moving forward, these reproductive cyclicity data can be applied to the advancement of assisted reproductive methodology for lions. Moreover, the development of a contraception method with a shorter duration of effectiveness and minimal long-term health impacts as well as placement of Suprelorin implants in locations that facilitate easy removal would provide a viable option for managing both *in situ* and *ex situ* lion population based on carrying capacity.

## Supporting Information

S1 Dataset Raw data for study animals (N = 38). (XLSX)

S1 Table Demographic information on study animals (N = 38). (XLSX)
